# Identification of serum proteins and multivariate models for diagnosis and therapeutic monitoring of lung cancer

**DOI:** 10.18632/oncotarget.14782

**Published:** 2017-01-21

**Authors:** Rong Ma, Heng Xu, Jianzhong Wu, Ashok Sharma, Shan Bai, Boying Dun, Changwen Jing, Haixia Cao, Zhuo Wang, Jin-Xiong She, Jifeng Feng

**Affiliations:** ^1^ Clinical Cancer Research Center, Jiangsu Cancer Hospital, Nanjing Medical University Affiliated Cancer Hospital Cancer Institute of Jiangsu Province, Nanjing, Jiangsu, 210009, China; ^2^ Jiangsu Province Institute of Materia Medica, Nanjing Tech University, Nanjing, 211816, China; ^3^ Center for Biotechnology and Genomic Medicine, and Department of Obstetrics and Gynecology, Medical College of Georgia, Augusta University, Augusta, GA, 30912, USA

**Keywords:** tumor antigens, NSCLC, inflammation, biomarkers, therapeutic response

## Abstract

Lung cancer is one of the most prevalent cancers and has very poor treatment outcome. Biomarkers useful for screening and assessing early therapeutic response may significantly improve the therapeutic outcome but are still lacking. In this study, serum samples from 218 non-small cell lung cancer (NSCLC) patients, 34 small cell lung cancer (SCLC) patients and 171 matched healthy controls from China were analyzed for 11 proteins using the Luminex multiplex assay. Eight of the 11 proteins (OPN, SAA, CRP, CYFRA21.1, CEA, NSE, AGP and HGF) are significantly elevated in NSCLC and SCLC (*p* = 10^−5^−10^−59^). At the individual protein level, OPN has the best diagnostic value for NSCLC (AUC = 0.92), two acute phase proteins (SAA and CRP) have AUC near 0.83, while CEA and CYFRA21.1 also possess good AUC (0.81 and 0.77, respectively). More importantly, several three-protein combinations that contain OPN and CEA plus one of four proteins (CRP, SAA, CYFRA21.1 or NSE) have excellent diagnostic potential for NSCLC (AUC = 0.96). Four proteins (CYFRA21.1, CRP, SAA and NSE) are severely reduced and three proteins (OPN, MIF and NSE) are moderately decreased after platinum-based chemotherapy. Therapeutic response index (TRI) computed with 3–5 proteins suggests that approximately 25% of the NSCLC patients respond well to the therapy and TRI is significantly correlated with pre-treatment protein levels. Our data suggest that therapeutic response in NSCLC patients can be effectively measured but personalized biomarkers may be needed to monitor different subsets of patients.

## INTRODUCTION

Lung cancer (LC) has a 5-year survival rate of approximately 15% and is one of the leading causes of all cancer-related deaths worldwide [1]. Although there has been improvement in early detection and treatment, the prognosis is still poor for lung cancer patients [2, 3]. Biomarkers that allow early diagnosis, guidance of therapeutic selection and/or early assessment of therapeutic outcome should improve care for lung cancer patients. Several widely known cancer antigens including cytokeratin 19 fragment (CYFRA21-1), carcinoembryonic antigen (CEA), neuron-specific enolase (NSE) have been found elevated in some lung cancer patients [4–7].

Chronic inflammation plays an important role in tumorigenesis. Inflammatory proteins may be increased by tumor growth which induces an inflammatory microenvironment [8, 9]. Thus, inflammatory proteins can potentially serve as biomarkers for cancer diagnosis, prognosis and recurrence. Various types of cancers have been associated with serum amyloid A (SAA), C-reactive protein (CRP) and α1-acid glycoprotein (AGP). These non-specific, acute-phase proteins are secreted in response to various cytokines including IL-1, IL-6 and TNF-α [10–12]. SAA and CRP are elevated in the serum of various cancers [13–18] including lung cancer [19, 20]. Both SAA and CRP also possess some prognostic potential for predicting survival of lung cancer patients [19, 20]. However, these proteins are elevated in a variety of disease conditions and are not sufficient for diagnostic or prognostic purposes by themselves.

Osteopontin (OPN) plays a critical role in many biological processes including tumor progression, metastasis and angiogenesis. OPN in the serum is elevated in different types of cancers [21–24] including lung cancer [25, 26] and patients with high serum OPN have poor survival [25–27]. Just like the other biomarkers discussed above, OPN by itself is not sufficient for clinical application.

Serum migration inhibitory factor (MIF) has been assessed as biomarker for lung cancer [28]. Hepatocyte growth factor (HGF) was found to be increased [29–31] in the serum of patients with lung cancer and high levels of serum HGF may be associated with poor survival [32]. However, another study found decreased serum HGF levels in lung cancer patients [33]. Serum E-Selectin was reported to be elevated in lung cancer patients [34, 35]. Growth-related oncogene (GRO) is expressed in many types of tumors. Serum GRO was found to be elevated in stage IV gastric cancer patients [36] although it has not been studied in lung cancer. A number of studies also attempted to discover combinations of serum proteins to achieve better diagnostic or prognostic value [7, 36–38]. Despite of improvement over single molecules, none of the reported combinations achieved sufficiently high specificity and sensitivity.

Assessing the therapeutic response soon after initiation of treatment is potentially of great importance to improve care for lung cancer patients. On one hand, stopping unsuccessful treatment can allow patients to consider different treatment options and on the other hand it avoids unnecessary side effects caused by unresponsive therapies. Biomarkers measured both before treatment or soon after treatment may be used for this purpose and have been target for development for many cancers [36, 39–41] and in limited studies for lung cancer [42–44]. However, clinically actionable biomarkers have yet to be developed.

In this study, we analyzed eleven serum proteins in a large panel of NSCLC patients and healthy controls as well as a small number of SCLC patients. We discovered several combinations of multiple proteins that can be used for NSCLC diagnosis or assessment of response to therapies.

## RESULTS

### Serum protein changes in lung cancer

**Figure 1:**
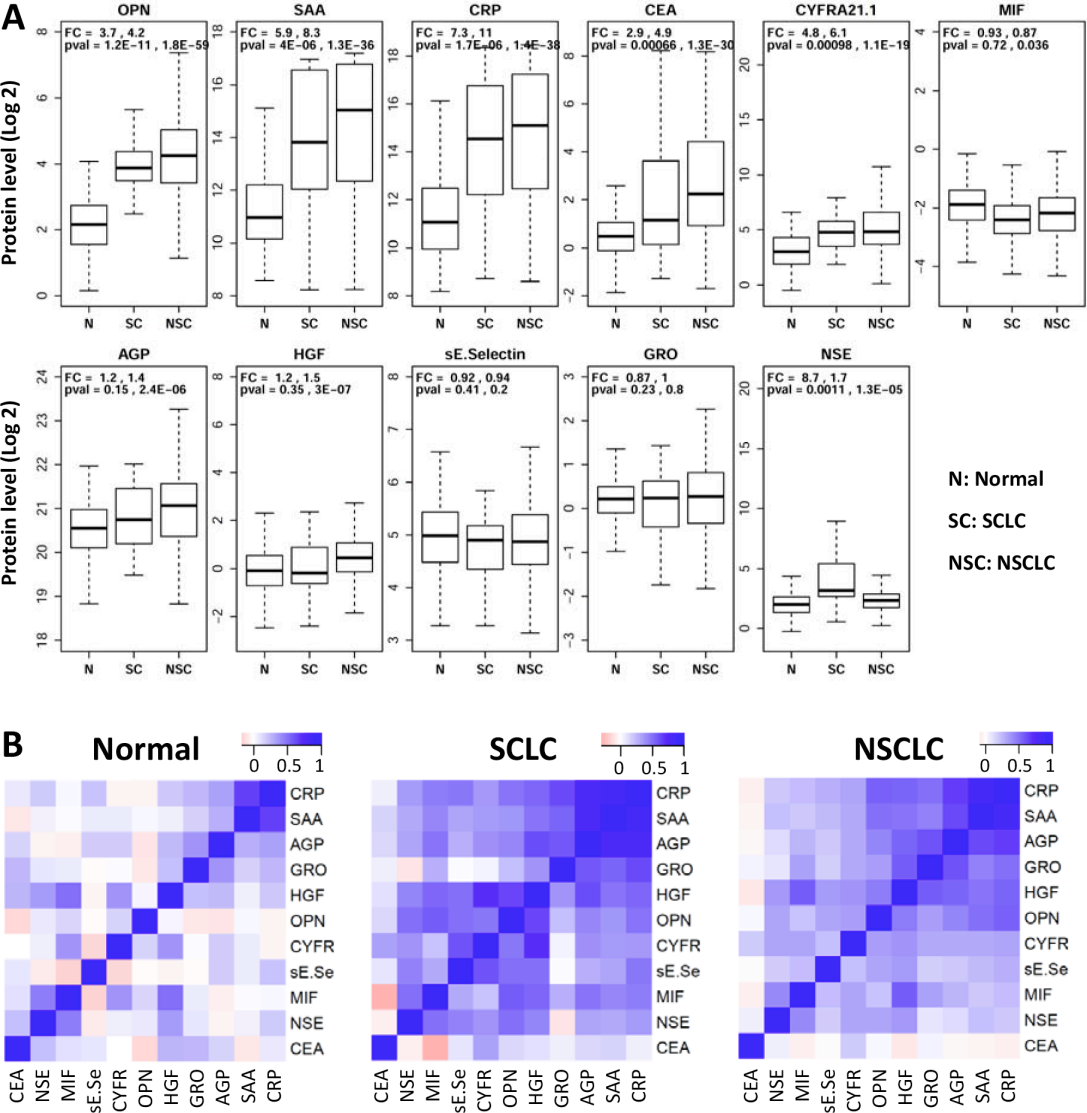
Distribution of serum protein levels measured in pre-treatment samples from 171 normal (N), 34 small cell (SC) and 218 non-small cell (NSC) lung cancer patients (**A**) Each plot represents distribution of protein levels on log 2 scale in three different groups. Fold change (FC) and *p*-values (pval) are shown on top of these plots (first value: SCLC *vs* Normal Controls; second value: NSCLC *vs* Normal Controls). (**B**) Heatmaps representing pair-wise correlations between levels of 11 proteins in three separate groups of subjects. Protein orders are based on clustering results and dendrograms are not shown here.

Eleven candidate proteins (CEA, CYFRA21.1, MIF, AGP, HGF, E-selectin, GRO, OPN, SAA, CRP, and NSE) were analyzed in serum samples from 218 NSCLC patients, 34 SCLC patients and 171 normal controls using Luminex multiplex assays. Figure 1A presents the raw data as box plots. Five of the eleven proteins (CEA, CYFRA21.1, OPN, SAA and CRP) were significantly increased in both NSCLC and SCLC patients compared to controls. The mean level of OPN is about 4-fold higher in both NSCLC and SCLC patients than controls (*p* < 10^−59^, and *p* < 10^−11^). The mean SAA level is more than 5-fold higher in NSCLC and SCLC patients than controls (*p* <10^−36^ and *p* < 10^−6^) and the mean CRP level is increased in patients by more than 7-fold (*p* < 10^−37^ and *p* < 10^−5^). The mean CEA level is 4.9-fold and 2.9-fold higher in NSCLC and SCLC, respectively (*p* < 10^−29^ and *p* < 0.001). The mean CYFRA21.1 level is 6.1-fold and 4.8-fold higher in NSCLC and SCLC, respectively (*p* < 10^−18^ and *p* < 0.001). However, MIF, AGP, HGF, sE-selectin, and GRO are not significantly different or are only marginally different in NSCLC or SCLC patients compared to controls. The only major difference between SCLC and NSCLC is NSE, which has an 8.7-fold increase in SCLC (*p* = 0.0011) but only 1.6-fold increase in NSCLC (*p* = 0.000013) compared to controls.

To exclude the possibility that differences between patients and controls are caused by confounding variables, logistic regression was performed using protein concentration as dependent variable and sex and age as covariates. NSCLC is significantly associated with eight of the eleven proteins after adjusting for age and sex (Table 1A), suggesting that the observed associations are not due to the examined covariates. Five of the eight proteins showing significant changes in NSCLC are also significantly different in SCLC (Table 1B) after adjusting for age and sex. Furthermore, NSE is increased in SCLC patients (OR = 2.4, P_adj_ = 0.01) but not in NSCLC (OR = 1.3, P_adj_ = 0.08). The comparison of protein levels in lung cancer patients with different stages (Stage-I and II: *n* = 19; Stage-III: *n* = 32; Stage-IV: *n* = 122) is presented in Supplementary Figure S1. CRP and CEA showed a small difference between early stage patients (stage I + II) and late stage patients (III + IV) but the difference is marginally significant before adjusting for multiple tests and not different after adjusting for multiple tests. Furthermore, the vast majority of the patients in this study are late stage patients and therefore stage is unlikely a major confounding factor in our studies.

**Table 1:** Logistic regression analyses of serum proteins in NSCLC and SCLC patients

NSCLC vs N	Unadjusted			Adjusted by Age & Sex		
Protein	OR	(95% CI)	*P*-value	OR	(95% CI)	*P*-value
OPN	7.00	(4.866–10.663)	1.66E-22	5.60	(3.734–9.018)	1.43E-14
SAA	1.81	(1.610–2.059)	2.65E-21	1.84	(1.574–2.201)	6.64E-13
CRP	1.73	(1.549–1.953)	1.36E-20	1.83	(1.567–2.173)	3.74E-13
CEA	2.18	(1.820–2.681)	2.51E-15	1.92	(1.544–2.443)	2.47E-08
CYFRA21.1	1.67	(1.454–1.939)	3.19E-12	1.72	(1.444–2.096)	1.12E-08
MIF	0.60	(0.456–0.766)	8.65E-05	0.61	(0.429–0.865)	6.05E-03
AGP	1.96	(1.425–2.746)	5.53E-05	2.63	(1.646–4.390)	1.04E-04
HGF	1.51	(1.220–1.902)	2.71E-04	1.50	(1.143–1.997)	4.14E-03
sE-Selectin	0.87	(0.649–1.162)	3.45E-01	1.15	(0.775–1.710)	4.91E-01
GRO	1.01	(0.714–1.439)	9.42E-01	1.01	(0.615–1.669)	9.56E-01
NSE	1.39	(1.167–1.696)	5.67E-04	1.28	(0.993–1.726)	8.14E-02
**SCLC vs N**	**Unadjusted**			**Adjusted by Age & Sex**		
**Protein**	**OR**	**(95% CI)**	***P*-value**	**OR**	**(95% CI)**	***P*-value**
OPN	11.65	(5.614–29.537)	4.55E-09	6.54	(2.838–19.768)	1.14E-04
SAA	1.74	(1.442–2.143)	3.60E-08	1.82	(1.383–2.507)	6.79E-05
CRP	1.74	(1.449–2.155)	3.22E-08	1.66	(1.273–2.277)	5.48E-04
CEA	1.87	(1.434–2.555)	1.76E-05	1.64	(1.104–2.613)	2.06E-02
CYFRA21.1	1.71	(1.332–2.265)	8.12E-05	1.79	(1.232–2.795)	4.90E-03
MIF	0.68	(0.416–1.064)	1.08E-01	0.63	(0.338–1.173)	1.47E-01
AGP	1.65	(0.882–3.187)	1.26E-01	1.18	(0.470–3.048)	7.32E-01
HGF	1.09	(0.779–1.468)	6.03E-01	1.01	(0.663–1.564)	9.50E-01
sE-Selectin	0.87	(0.492–1.493)	6.15E-01	1.01	(0.474–2.179)	9.75E-01
GRO	0.64	(0.346–1.171)	1.48E-01	0.37	(0.126–1.004)	5.53E-02
NSE	2.47	(1.707–3.840)	1.19E-05	2.42	(1.411–5.309)	1.33E-02

We next examined whether serum protein levels are correlated with each other and the data are analyzed in three separate groups. To identify clusters of correlated proteins, the pair-wise correlation matrix is presented as a heatmap and subjected to hierarchical clustering (Figure 1B). The data indicate that only CRP and SAA are correlated in controls, while there are two subsets of correlated proteins in both lung cancer groups. The first correlated subset of proteins include CRP, SAA, AGP, GRO, HGF and OPN (average correlation coefficients are 0.17, 0.56 and 0.55 for normal controls, SCLC and NSCLC, respectively), while the second group of correlated proteins includes HGF, OPN, CYFR21.1, sE-selectin, MIF and NSE (average correlation coefficients are 0.14, 0.49 and 0.34 for normal controls, SCLC and NSCLC, respectively). Interestingly, CEA is not correlated with any other protein (average correlation coefficients 0.09, 0.08 and 0.003 for the three subject groups).

### Correlations with patient characteristics

Metastatic patients have significantly higher CEA than non-metastatic patients (FC = 2.1, *p <* 0.004) (Supplementary Figure S2). MIF is marginally lower in patients with metastasis than without metastasis (FC = 0.8, *p* = 0.1). No other significant differences were found.

### Diagnostic value for NSCLC

**Figure 2:**
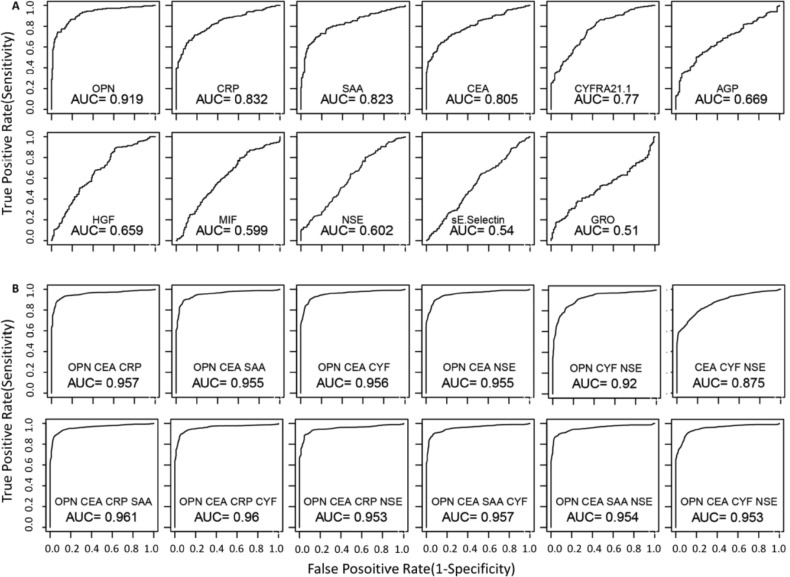
Receiving-operator-characteristics (ROC) curves that evaluate the ability to distinguish NSCLC patients from healthy controls Single proteins (**A**) and multi-protein models (**B**) were used for the analyses. For multi-protein models, linear discriminate analysis was performed using all possible combinations for 3 or 4 of the 6 top performing proteins (OPN, CEA, CRP, SAA, CYFRA 21-1 and NSE). The performance of each model was evaluated using leave one out cross validation method. The utility of serum proteins as NSCLC biomarkers was evaluated using the area-under-curve (AUC) of the ROC curves for different models. ROC curves are only shown for the top performing models.

Area-under-the-curve (AUC) in receiver-operating-characteristic (ROC) curves were examined for each of the eleven proteins for their ability to separate NSCLC patients from controls. As shown in Figure 2A, some proteins have excellent but not perfect AUC values. The best proteins are OPN (AUC = 0.919), CRP (AUC = 0.832), SAA (AUC = 0.823) and CEA (AUC = 0.805). The cancer antigens CYFRA21.1 and NSE have AUC values of 0.77 and 0.60, respectively.

Subsequently, we examined AUC values using all combinations of three or four proteins consisting of OPN, CEA, CRP, SAA, CYFRA21.1 and NSE. As shown in Figure 2B, AUC for NSCLC can be improved by multiple combinations. Four best models with three proteins have near perfect AUC values (~0.96) and all four models contain both OPN and CEA, with a third protein being one of the other four proteins (NSE, CYFRA21.1, CRP, or SAA) (Figure 2B). However, no model with four proteins further improved the diagnostic value compared to the best three-protein models (Figure 2B).

Table 2 presents the sensitivity values for individual proteins as well as different models at four different specificity thresholds (90%, 95%, 99% and 100%). Among individual proteins, the best performing protein is OPN, which has a sensitivity of 78%, 72%, 58% and 29% at specificity of 90%, 95%, 99% and 100%, respectively. The performance for CEA, CRP and SAA are comparable but worse than OPN. At the highest specificity requirement (99% and 100%), four multivariate models with three proteins (OPN-CEA plus CYFRA21.1, NSE, CRP or SAA) have reached sensitivity levels of approximately 70% and 60% (Table 2), suggesting that these protein combinations possess excellent diagnostic potential.

**Table 2:** Sensitivity of individual and combinations of proteins at different specificity threshold for NSCLC

Protein	AUC	Sensitivity at specificity of				*p*-val
		90%	95%	99%	100%	
OPN	0.92 (0.91–0.93)	77.5	72.5	57.8	28.9	1.1E-45
CEA	0.80 (0.78–0.83)	56.4	50.5	41.3	35.8	5.2E-25
CRP	0.83 (0.81–0.85)	63.8	52.8	39.9	39.5	2.5E-29
SAA	0.82 (0.80–0.84)	63.8	56.0	30.7	21.1	7.5E-28
CYFRA21.1	0.77 (0.75–0.79)	42.4	35.9	25.8	24.9	6.5E-20
NSE	0.60 (0.57–0.63)	19.7	12.8	11.0	9.6	5.5E-04
AGP	0.67 (0.64–0.70)	36.5	30.5	14.0	13.0	5.8E-06
HGF	0.66 (0.63–0.68)	18.8	11.9	4.6	0.5	7.3E-08
MIF	0.60 (0.57–0.63)	17.0	4.10	1.8	0.0	8.1E-04
sE-Selectin	0.54 (0.51–0.57)	10.5	6.4	0.6	0.0	1.8E-01
GRO	0.51 (0.47–0.55)	20.5	17.5	7.5	2.0	7.9E-01
CEA OPN CRP	0.96 (0.95–0.96)	91.7	87.6	74.6	62.4	< E-300
CEA OPN NSE	0.96 (0.95–0.96)	90.3	81.0	70.1	63.0	< E-300
CEA OPN CYF	0.96 (0.95–0.96)	90.8	84.7	69.4	65.0	< E-300
CEA OPN SAA	0.96 (0.95–0.96)	90.3	85.3	62.7	60.2	< E-300
CEA OPN CRP SAA	0.96 (0.96–0.96)	91.7	88.1	67.0	61.0	< E-300
CEA OPN CRP CYF	0.96 (0.96–0.96)	92.0	88.4	68.4	64.0	< E-300
CEA OPN SAA CYF	0.96 (0.95–0.96)	91.0	87.3	69.9	61.5	< E-300
CEA OPN CRP NSE	0.95 (0.95–0.96)	91.1	88.6	69.0	66.7	< E-300
CEA OPN SAA NSE	0.95 (0.95–0.96)	90.7	87.5	69.3	62.6	< E-300
CEA OPN CYF NSE	0.95 (0.95–0.96)	89.0	77.9	69.4	65.1	< E-300

CYF: CYFRA21.1.

The ability of each protein to distinguish NSCLC patients from SCLC patients was also examined. As shown in Supplementary Figure S3, the best AUC value for individual proteins is 0.722 (NSE). Protein combinations could not improve the performance over single proteins. We did not evaluate the diagnostic potential for these proteins for SCLC due to the small sample size.

### Changes in response to therapy

**Figure 3:**
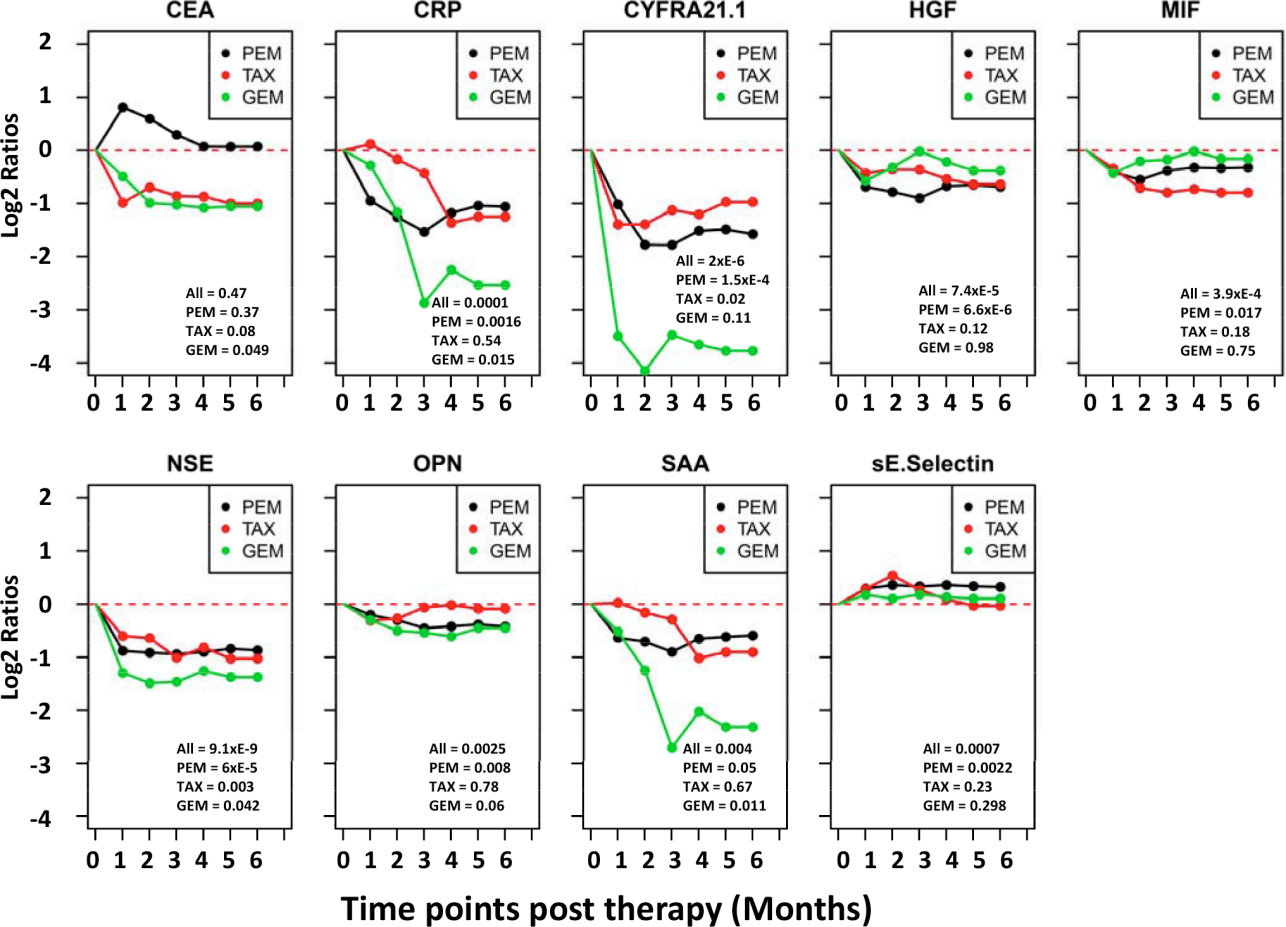
Trend lines of mean protein levels post therapy in three different treatment groups of NSCLC patients Subjects are divided into three treatment groups, which received platinum plus one of the three drugs: PEM = pemetrexed, TAX = taxane, GEM = gemcitabine. The time point before treatment is set as 0 time point and the mean protein levels before treatment in each group are set as zero for the group. For each of the six months after treatment, the protein levels of each patient is divided by the pretreatment level in that patient to calculate the post *vs* pre-therapy ratio, which is then Log 2 transformed. The Log 2 ratios of all patients in the treatment group are averaged and plotted. If data are missing for any time point for a patient, measurement at the previous time point is used for data analysis. Paired T test was used to compare each post treatment time point and the pretreatment time point for statistical significance. Values at lower-right corner of each chart are *p*-values for pre-therapy *vs* 3-months post therapy comparisons for three treatment groups. *P* values for the 4, 5 and 6 month time points are similar but not shown.

Serum samples before and post treatment were available from 68 NSCLC patients, all of whom received platinum-based therapies. The treatment can be grouped in three major categories based on the other treatment drugs. The first group included 43 patients treated with platinum and pemetrexed (PEM) and the second group had 17 patients treated with platinum and taxane (TAX), while the third group includes 8 patients that received platinum plus gemcitabine (GEM). For each individual patient, the ratios between protein levels post-treatment over pre-treatment were calculated for each protein. The mean ratios in each of the three treatment groups are presented in Figure 3. The data indicate that several proteins were reduced post treatment in all three treatment groups. Proteins with greater reductions after treatment include CYFRA21.1, NSE, CRP and SAA. CEA is very interesting because its mean level is reduced post treatment in the gemcitabine and taxane treatment groups but was actually increased in the pemetrexed treatment group.

**Figure 4:**
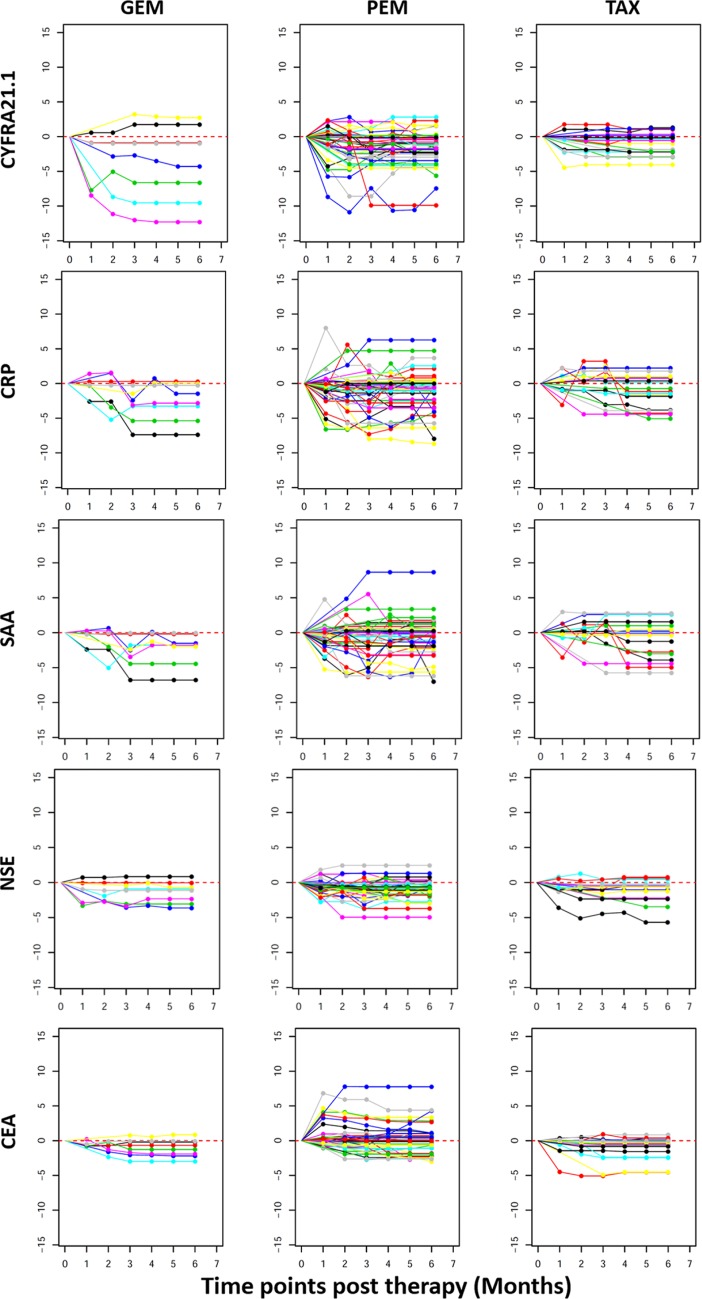
Line plots depicting post therapy changes in the protein levels in individual NSCLC patients Plotted on the Y axis are Log 2 ratios of post vs after treatment protein levels as explained in Figure 3 and X-axis plots the treatment time points. Plotted data are for individual patients (each represented by a line) to illustrate the variable response to treatment. Within each treatment group (GEM, PEM or TAX), each patient is represented by lines with the same color in plots for different proteins. If data is missing for any time point, measurement at the previous time point is used for data analysis.

Figure 4 shows the changes of individual patients in each of the three treatment groups. The first interesting observation is the great variability among different patients. For example, in the PEM group, CEA is greatly increased after treatment in some patients while some other patients have reduced levels. A subset of patients also showed dramatic reduction in the levels of CYFRA21.1, NSE, CRP and SAA. Furthermore, these four proteins are consistently changed or unchanged in most patients. Finally, there are higher percentages of patients with severe reduction in the GEM treatment compared to Taxane and PEM treatment.

**Figure 5:**
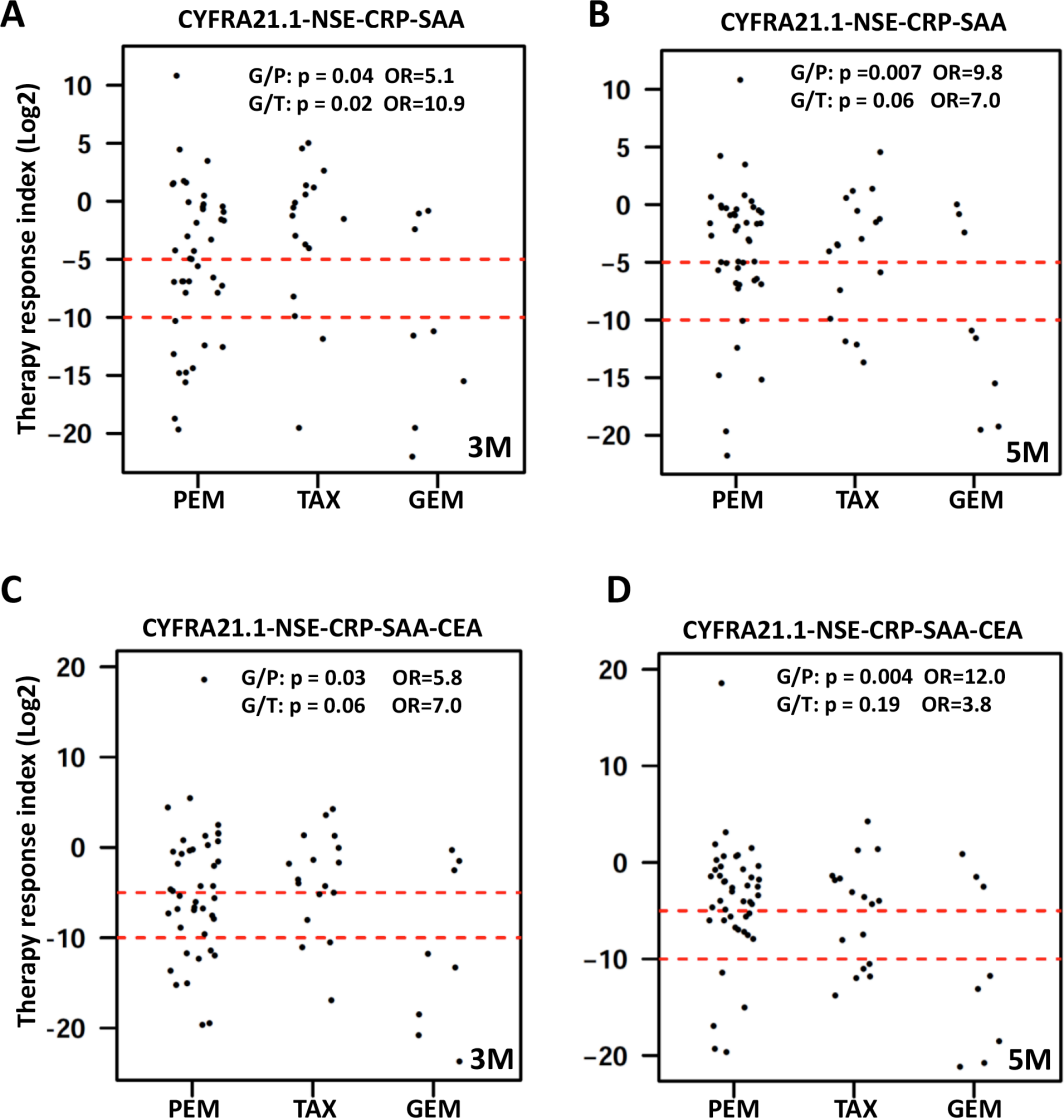
Dot plots of therapy response index (TRI) calculated for each patient TRI was calculated for each patient (represented by a dot) and time point as explained in the text. Presented here are representative dot plots for TRI calculated using four proteins (upper panel) or five proteins (lower panel) at the 3 month time point (left panels marked as 3 M) and the 5 month time point (right panels marked as 5 M). Data for other time points are similar and not shown here. Dashed red lines represent the cutoff values for good response (TRI < 2^−10^) and moderate response (TRI < 2^−5^). Differences in treatment response were compared between GEM and PEM treatment groups (G/P) and between GEM and TAX groups (G/T) by calculating the odds ratio (OR) and associated *p value* using TRI < 2^−10^ as cutoff for good response.

To assess the overall changes of serum protein profile, we computed a therapy response index (TRI). The ratio of post/pre-treatment concentration was calculated for each patient and each protein. TRI for each patient is the sum of the log 2 ratios for the proteins used for TRI calculation. TRI can be calculated using any number of proteins but we focused on four proteins (CYFRA21.1-CRP-SAA-NSE) and five proteins (4 proteins + CEA). TRI can also be computed for different time points post treatment. TRI is shown as dot plots for selected combinations of proteins and time points (Figure 5) and similar data were obtained for other time points and other protein combinations. We also examined the percentage of patients with different levels of TRI. Based on the distribution of TRI in the entire cohort, we selected two thresholds to examine the treatment outcomes. A TRI < 2^−10^ (or 1/1028) is considered as good response, while a TRI of 2^−5^−2^−10^ is considered moderate response. At the 3 month post-treatment time point, 27% of the patients had a TRI < 2^−10^ and 17.5% of the patients had a TRI of 2^−5^−2^−10^, while 55.5% of the patients had only modest reduction, unchanged or moderate increase (TRI > 2^−5^) (Figure 5). Since these serum proteins are increased in NSCLC patients compared to controls, their reduction in response to therapy should indicate reduction of tumor burden after therapy and the degree of reduction may be a reflection of the success of therapy.

We next examined the potential difference in treatment outcome between the three treatment groups (Figure 5). Using TRI of 2^−10^ as indication of good response to therapy, 62.5% of the patients receiving gemcitabine achieved good response compared to 26% and 13% for the pemetrexed groups at 3 and 5 months post therapy (OR = 5.1, *p* = 0.04; and OR = 10.8, *p* = 0.01, respectively). In the taxane group, 12% and 18% of the patients achieved good response at 3 and 5 months, which are significantly different from the gemcitabine treatment group (OR = 10.8, *p* = 0.01 and OR = 7.0, *p* = 0.06), respectively) but not significantly different from the pemetrexed group. These results may suggest that platinum plus gemcitabine is a better treatment for NSCLC.

### Prediction of therapy response using proteins measured before therapy

**Figure 6:**
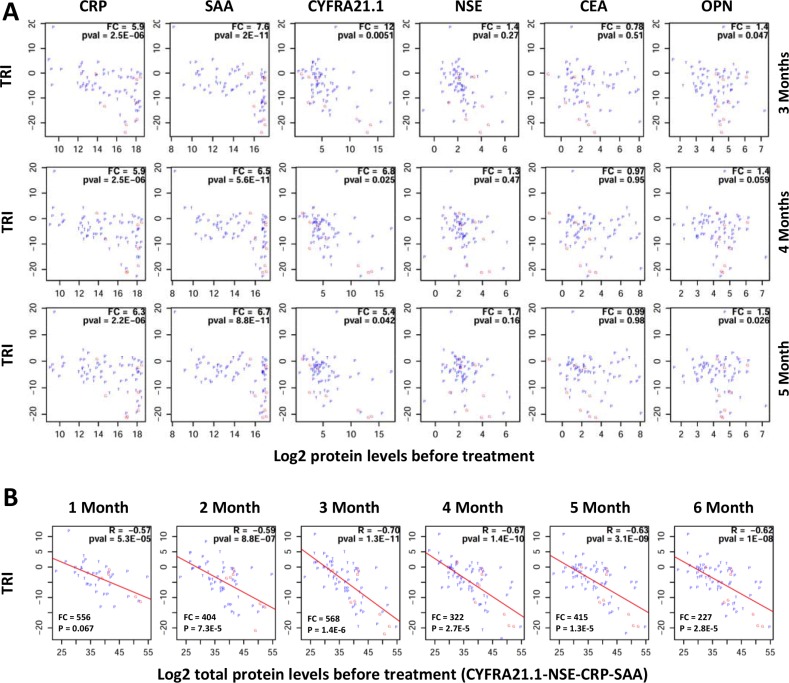
Correlation between pre-treatment protein levels and therapy response index (TRI) TRI was calculated using a combination of four proteins (FRA21.1-CRP-SAA-NSE). Each patient is represented by a letter (P, G or T) corresponding to their treatment groups (PEM, GEM or TAX). (**A**) Plots showing correlations between TRI (y-axis) and pre-therapy protein levels for individual proteins (x-axis). FC (fold change) is the ratio of mean protein levels for patients with TRI < 2^−10^ (good response) over mean protein level for patients with TRI ≥ 2^−10^. (**B**) Plots showing correlations between TRI (y-axis) and the sum of protein levels for four proteins (CYFRA21.1, NSE, CRP and SAA) at the pre-therapy time point (x-axis). FC (fold change) is the ratio of mean total protein levels for patients with TRI < 2^−10^ (good response) over mean total protein level for patients with TRI ≥ 2^−10^. R: correlation coefficient; pval = *p value*. Treatment time points are shown on top of the correlations plots.

We also examined whether protein changes in response to therapy can be predicted by the protein levels before treatment. Figure 6A plots the correlation between TRI and the pre-treatment levels for individual proteins. These results suggest that patients with good response tend to have much higher protein levels for CRP, SAA and CYFRA21.1. Although patients with the highest protein levels tend to respond better, not all patients with high expression respond well to treatment.

Subsequently, we examined whether the response to treatment and the total levels of multiple proteins. Surprisingly, very high correlations were observed between TRI and the total protein levels of multiple proteins. Representative data for four proteins (CRP-SAA-CYFRA21.1-NSE) are shown in Figure 6B. The data suggest again that patients with higher protein levels have better TRI.

## DISCUSSION

LC is a cancer with high mortality and morbidity in China and many other countries in the world and LC patients can be treated with surgery, chemotherapy and radiotherapy [45]. A number of studies have searched for serum protein biomarkers that can distinguish LC from controls and various associations have been suggested in the literature [46]. However, no biomarker reported so far is of great clinical interest due to the poor specificity and sensitivity. Therefore, the search for better biomarkers, either individual molecules or specific combinations of molecules, continues.

In this study, we analyzed 11 serum proteins in a relatively large cohort of LC patients and healthy controls and identified six proteins (OPN, CEA, NSE, CYFRA21.1, CRP, and SAA) that, in combination, may be of great clinical interest for NSCLC. The first potential application of these six proteins is related to NSCLC diagnosis. Among individual proteins, OPN is the best biomarker for diagnosing NSCLC (AUC = 0.919) in this study. This AUC value is also better than any individual proteins reported for NSCLC in the literature. OPN has been reported to be elevated in NSCLC serum and tissues and is associated with poor survival [25, 26]. In this study, NSCLC patients have 4.2 times higher OPN and SCLC patients have 3.7 times higher OPN than controls (*p <* 10^−58^). The AUC value for NSCLC is 0.919, indicating a good diagnostic potential.

The next two best performing individual proteins for NSCLC are CRP (AUC = 0.832) and SAA (AUC = 0.823), two inflammatory proteins. It has been reported that CRP is higher in NSCLC patients compared to healthy controls but has modest prognostic value for survival in NSCLC patients [19, 20]. SAA was found to be increased by 14-fold in NSCLC patients compared to controls [47] and higher SAA level was also associated with poor survival in NSCLC [48]. This study with 218 NSCLC patients and 171 healthy controls convincingly shows significantly elevated CRP and SAA in LC patients compared to healthy controls. CRP is 11.0 times higher in NSCLC and 7.3 times higher in SCLC patients compared to controls. SAA is 8.3 times higher in NSCLC and 5.9 times higher in SCLC patients compared to controls. The inflammatory response to tumor and expression of CRP and SAA in tumor cells [49] may account for the coordinated elevation of SAA and CRP in cancer patients.

Three widely reported tumor antigens (CEA, NSE and CYFRA21.1) may be of diagnostic value for NSCLC although individually their AUC is modest (0.805, 0.77 and 0.60, respectively). CEA is 4.9 times higher in NSCLC and 2.9 times higher in SCLC. CYFRA21.1 is 6.1 times higher in NSCLC and 4.8 times higher in SCLC. NSE is only 1.7 times higher in NSCLC but 8.7 times higher in SCLC. NSE is indeed the only protein that is significantly higher in SCLC than NSCLC and may be an excellent biomarker for SCLC. The AUC values observed in this study for these three cancer antigens are in lines with findings reported in other studies [50].

One of the most important discoveries in this study is that combinations (models) with multiple proteins can significantly improve the performance of serum proteins for diagnosis of NSCLC. Only six proteins with better performance based on individual proteins (OPN, CEA, CRP, SAA, CYFRA 21-1 and NSE) were used in the combination analyses. We evaluated all 30 combinations with three proteins in each model and all 15 models with four proteins in each model. Four models, each with three proteins, reached an outstanding AUC near 0.96. This is the best AUC achieved for LC to our knowledge. Interestingly, all four models contain OPN and CEA, with a third protein being CYFRA21.1, NSE, CRP or SAA. Although four models have similar performance, we believe that the models with tumor antigens (OPN-CEA-CYFRA21.1 and OPN-CEA-NSE) may be more appropriate than the models with inflammatory proteins (OPN-CEA-CRP and OPN-CEA-SAA) because CRP and SAA are elevated in many diseases including other cancers. Therefore, the combinations of four serum proteins (OPN, CEA, CYFRA21.1 and NSE) may be excellent biomarkers for NSCLC diagnosis. Their potential for NSCLC screening should also be evaluated in future studies.

The second most important finding in this study is related to biomarkers that can assess the response to therapies and indication of disease recurrence. Previous studies have shown that poor outcome is predicted by high levels, slower and incomplete decline in CEA, CYFRA 21-1 and nucleosomal DNA [42–44]. Although these biomarkers are of some help to the management of individual patients, these tools are not ideal because the differences between the responder groups and non-responder groups are usually small and overlap between the groups is quite significant. In this study, we examined the reduction of serum proteins by calculating the ratios between post- and pre-therapy concentrations and identified five proteins (CYFRA21.1, CEA, NSE, CRP and SAA) that are severely reduced in subsets of NSCLC patients in response to therapy. In many cases, multiple proteins are coordinately reduced by therapy. Therefore, we computed for each patient a therapy response index (TRI). TRI clearly identified a subset, approximately a quarter, of the NSCLC patients that have dramatically reduced protein levels (TRI < 2^−10^). These patients responded very well to therapy as measured by the examined serum proteins that are known to be implicated in NSCLC. Unfortunately, survival data are not available for these patients to correlate TRI and patient survival.

Since prognostic biomarkers for patient response to therapy can improve patient care, we examined whether serum protein levels can predict therapeutic response in NSCLC. It was interesting to find out that almost all patients with great TRI have high levels of CYFRA21.1, CRP and SAA as well as slightly higher levels of NSE and OPN. Although the response to treatment is clearly associated with individual protein levels, the total protein levels of multiple proteins showed very good correlation with TRI. However, the clinical implication of these findings requires further investigation. It is now unclear whether the reduction of these proteins translates into long term survival benefit. Since these proteins are clearly NSCLC biomarkers, we believe that their reduction by therapy should be correlated with patient outcome. It is also unknown why TRI is correlated with pre-treatment protein levels. One likely possibility is that the therapeutic response can be measured more effectively with biomarkers that are more highly expressed in the patients. This is a logic hypothesis and suggests that different biomarkers will be needed to monitor therapeutic outcome for different subsets of patients, the essence of personalized or precision medicine.

## MATERIALS AND METHODS

### Human subjects and serum samples

The selection criteria for patients with lung cancer were as follows: 1) pathologically confirmed patients (the diagnoses in all patients were confirmed each time by microscopic examination of the material obtained during bronchoscopy, biopsy, or surgery); 2) patients had no history of other carcinomas. A total of 218 NSCLC patients, 34 SCLC patients in Jiangsu Cancer Hospital between October 2011 and December 2012 and 171 healthy control subjects from Nanjing were used in the present study. Blood samples were collected from patients at the time of diagnosis and before any treatment (surgery and/or chemotherapy). Furthermore, blood samples from patients treated with surgery and/or chemotherapy were also collected monthly. Samples were centrifuged for 10 min at 3,000 rpm at 4°C, and serum was subsequently frozen at −80°C until use. This study has been approved by the human subject ethics committee of the Jiangsu Cancer Hospital and informed consent signed by the study subjects.

### Luminex assays

Luminex assays for all proteins were obtained from Millipore (Millipore Inc., Billerica, MA, USA). The assays were performed according to the manufacturer's instructions. Briefly, serum samples were incubated with antibody-coated microspheres, followed by biotinylated detection antibody. Proteins were detected by incubation with phycoerythrin-labeled streptavidin and the resultant bead immuno-complexes were read on a FLEXMAP3D (Luminex, TX, USA) with the following instrument settings: events/bead: 50, minimum events: 0, Flow rate: 60 ul/min, Sample size: 50 ul, discriminator gate: 8000–13500. Median fluorescence intensity (MFI) was collected and used for calculating protein concentration.

### Statistical analysis

Protein concentrations were estimated using a regression fit to the standard curve with known concentration included on each plate using a serial dilution series. The concentrations were logarithmically transformed prior to all statistical analyses to achieve normal distribution. The comparisons for 3 groups (N, SCLC, and NSCLC) were made by ANOVA followed by pair-wise group comparisons using *t*-tests (Figure 1A). To examine the relationships between disease status and serum protein levels, logistic regression was used by including age and sex as co-variates (Table 1). To examine the correlation between levels of 11 proteins, the pairwise correlations were computed using Pearson correlation in controls, SCLC and NSCLC groups separately. Clustering and visualization of correlation matrix was performed using hierarchical clustering method and heatmap (Figure 1B). The diagnostic power of individual proteins and their combinations to differentiate controls and NSCLC patients was assessed using the area under the curve (AUC) of the receiver operating characteristic (ROC) curves (Figure 2). Sensitivity values of individual and combinations of proteins at different specificity thresholds (90%; 95%; 99%; 100%) were computed (Table 2). This ROC analysis was not performed for SCLC patients due to smaller sample size. Log_2_ ratios of protein levels post- *vs* pre-treatment were computed at six different time points (1–6 month post-treatment). If data are missing for any time point, measurement at the previous time point was used for data analysis. For pre-post comparisons a paired *t-test* was used to compute *p*-values for individual treatment groups and all patients combined (Figure 3). The trend of pre- and post-treatment protein levels in individual patients was visualized using line plots (Figure 4). Therapy Response Index distribution was plotted for each patient at two time points (3-months and 5-months post treatment). The comparison of TRI values in three treatment groups (PEM, TAX, and GEM) was made using fisher's exact test (Figure 5). To examine the relation between the therapy response and protein levels before treatment, we computed Pearson correlation coefficient between TRI values and individual protein levels of CRP, SAA, CYFRA21.1, NSE, CEA and OPN. Correlation of TRI values with total protein levels before treatment was also computed (Figure 6). All statistical analyses were performed using the R language and environment for statistical computing (R version 2.15.1; R Foundation for Statistical Computing; www.r-project.org).

## SUPPLEMENTARY MATERIALS FIGURES


