# Supplementary material for: Identification of serum proteins and multivariate models for diagnosis and therapeutic monitoring of lung cancer
Source: Oncotarget. 2017 Jan 21;8(12):18901–13. doi: 10.18632/oncotarget.14782 (PMC5386656; doi:10.18632/oncotarget.14782)
Supplement: Supplementary file 1 [file oncotarget-08-18901-s001.pdf]

Identification of serum proteins and multivariate models for diagnosis and therapeutic monitoring of lung cancer

Supplementary Materials

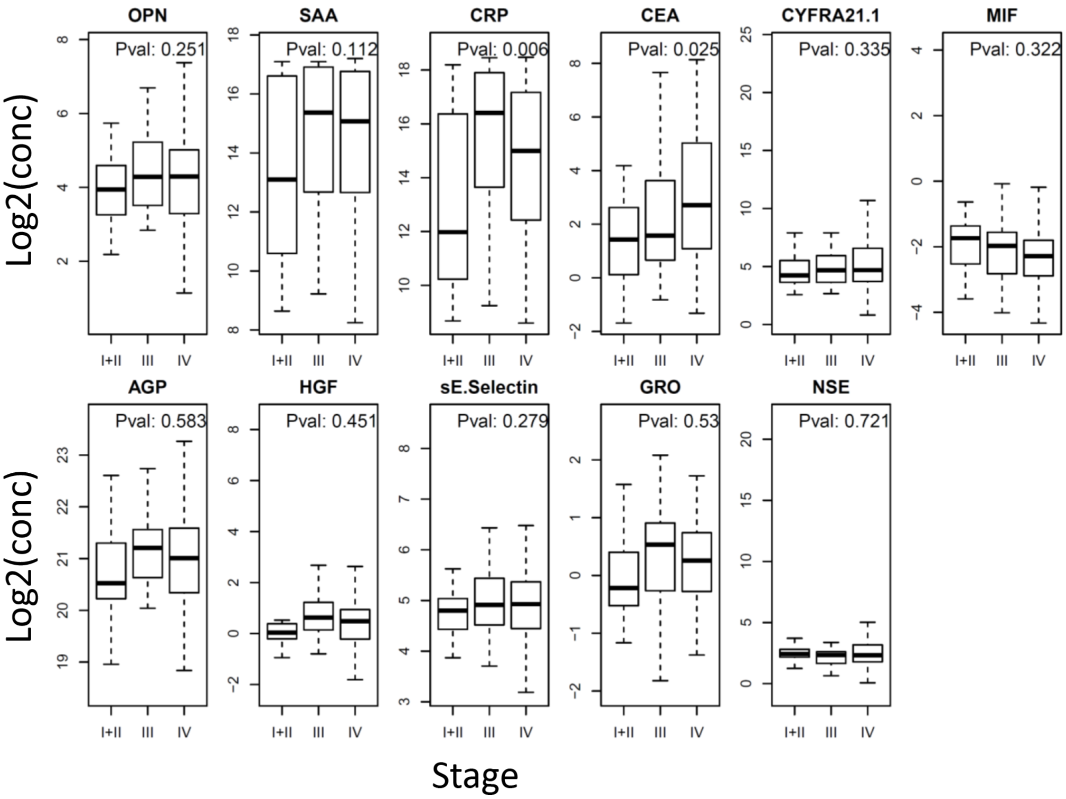

Supplementary Figure S1: Comparison of protein levels in different TNM stage lung cancer patients (Stage-I and II: *n* = 19; Stage III: *n* = 32; Stage IV: *n* = 122).

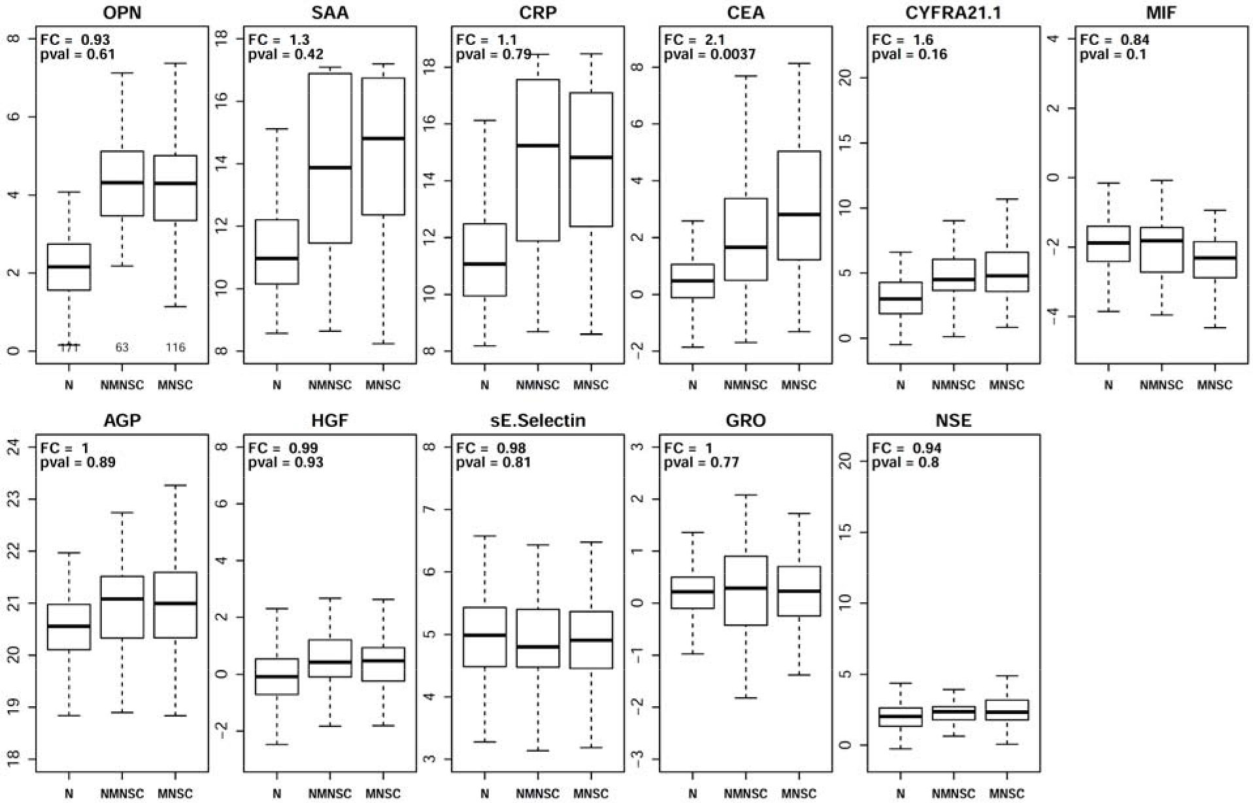

Supplementary Figure S2: Difference between patients with and without distant metastasis. Normal controls (N), NSCLC patients without distant metastasis (NMNSC) and NSC-LC patients with distant metastasis (MNSC) were compared. FC = fold change.

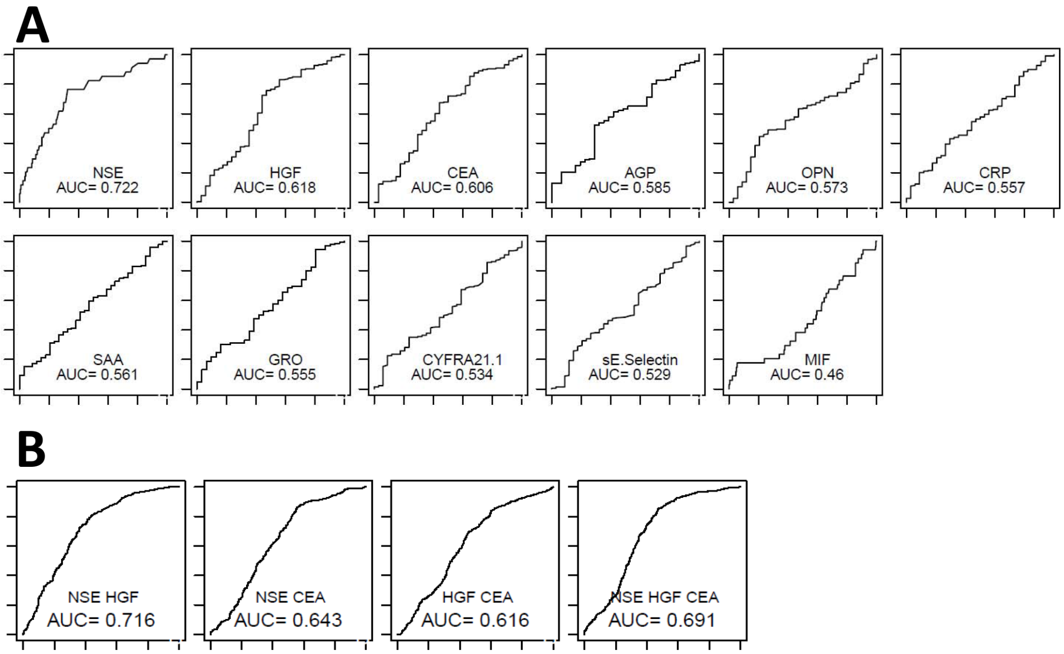

Supplementary Figure S3: ROC and AUC to distinguish NSCLC from SCLC by individual proteins (**A**) or combinations of proteins (**B**).
